# Different hemodynamic factors cause the occurrence of superior mesenteric atherosclerotic stenosis and superior mesenteric artery dissection

**DOI:** 10.3389/fcvm.2023.1121224

**Published:** 2023-04-18

**Authors:** Junhao Mei, Wei Ding, Haiyang Yu, Xi Zhao, Haoran Xu, Kai Wang, Zhongzhi Jia, Benling Li

**Affiliations:** ^1^ Department of Interventional and Vascular Surgery, The Affiliated Changzhou Second People's Hospital of Nanjing Medical University, Changzhou, China; ^2^ Department of Interventional Radiology, The Affiliated Wuxi People's Hospital of Nanjing Medical University, Wuxin, China; ^3^ Central Research Institute, United Imaging Healthcare, Shanghai, China; ^4^ Department of Pathology, The Affiliated Changzhou Second People’s Hospital of Nanjing Medical University, Changzhou, China; ^5^ Department of Cardiology, The Affiliated Changzhou Second People's Hospital of Nanjing Medical University, Changzhou, China

**Keywords:** mesenteric artery, atherosclerotic stenosis, dissection, computational fluid dynamics, histopathologic

## Abstract

**Objective:**

To compare the hemodynamic factors involved in the occurrence of superior mesenteric atherosclerotic stenosis (SMAS) and superior mesenteric artery (SMA) dissection (SMAD).

**Methods:**

Hospital records were searched to identify consecutive patients who were diagnosed with SMAS or SMAD between January 2015 and December 2021. A computational fluid dynamics (CFD) simulation method was used to assess the hemodynamic factors of the SMA in these patients. Histologic analysis was also performed on SMA specimens obtained from 10 cadavers, and scanning electron microscopy was used to evaluate collagen microstructure.

**Results:**

A total of 124 patients with SMAS and 61 patients with SMAD were included. Most SMASs were circumferentially distributed at the SMA root, whereas the origin of most SMADs was located on the anterior wall of the curved segment of the SMA. Vortex, higher turbulent kinetic energy (TKE), and lower wall shear stress (WSS) were observed near plaques; higher TKE and WSS were seen near dissection origins. The intima in the SMA root (388.5 ± 202.3 µm) was thicker than in the curved (243.8 ± 100.5 µm; *p *= .007) and distal (183.7 ± 88.0 µm; *p* < .001) segments. The media in the anterior wall (353.1 ± 37.6 µm) was thinner than that in the posterior wall (473.7 ± 142.8 µm; *p *= .02) in the curved segment of the SMA. The gaps in the lamellar structure in the SMA root were larger than in the curved and distal segments. The collagen microstructure was more substantially disturbed in the anterior wall than in the posterior wall in the curved segment of the SMA.

**Conclusion:**

Different hemodynamic factors in different portions of the SMA are related to local pathological changes in the SMA wall and may lead to the occurrence of SMAS or SMAD.

## Introduction

Superior mesenteric atherosclerotic stenosis (SMAS) and superior mesenteric artery (SMA) dissection (SMAD) are two of the most common causes of mesenteric ischemia (1). With the development of imaging techniques, SMAD, once considered as a rare disease, is increasingly diagnosed in recent years (2). However, these disease processes differ in terms of their characteristics and the pathological changes involved. For instance, SMAS is always located at the root of the SMA (3, 4), whereas SMAD occurs at the convex surface of the SMA trunk, 1 to 3 cm away from the root (5, 6). Studies have speculated that this difference in location may be related to the hemodynamic factors of the SMA (7–9). However, individual hemodynamic parameters in patients with SMAS or SMAD have not been analyzed. Different from aortic dissection, patients with SMAS and SMAD are mostly treated by conservative or endovascular therapy (10). Therefore, it is difficult to obtain pathological tissue specimens of patients with these two diseases, and data regarding the exact relationship among these hemodynamic factors, pathologic changes, and the disease characteristics of SMAS and SMAD are still limited. The pathogenesis of SMAS and SMAD is poorly understood at present.

In this study, we therefore sought to explore the role of hemodynamic factors in the occurrence of SMAS and SMAD using personalized SMA models and patient-specific boundary conditions based on a computational fluid dynamics (CFD) simulation method. We also sought to identify the pathologic changes caused by hemodynamic factors that may be associated with the occurrence of SMAS and SMAD.

## Materials and methods

### Patients

This retrospective study was approved by our institutional review board with a waiver of informed consent. Our hospital records were searched to identify consecutive patients who had been diagnosed with SMAS or SMAD between January 2015 and December 2021 [*via* contrast-enhanced computed tomography (CT)]. SMAS was diagnosed if wide base calcification or a filling defect with low density was seen on CT after contrast agent injection (11). SMAD was diagnosed if the true and false lumens and the intima flap between them were seen on CT, as outlined previously (12). Patients were excluded from the study if they had aortic or any other artery dissection; if they had previously undergone SMA surgery; or if other factors affecting the aortomesenteric angle, such as scoliosis or intra-abdominal tumor, were present.

### Image acquisition and lesion localization

**Figure 1:**
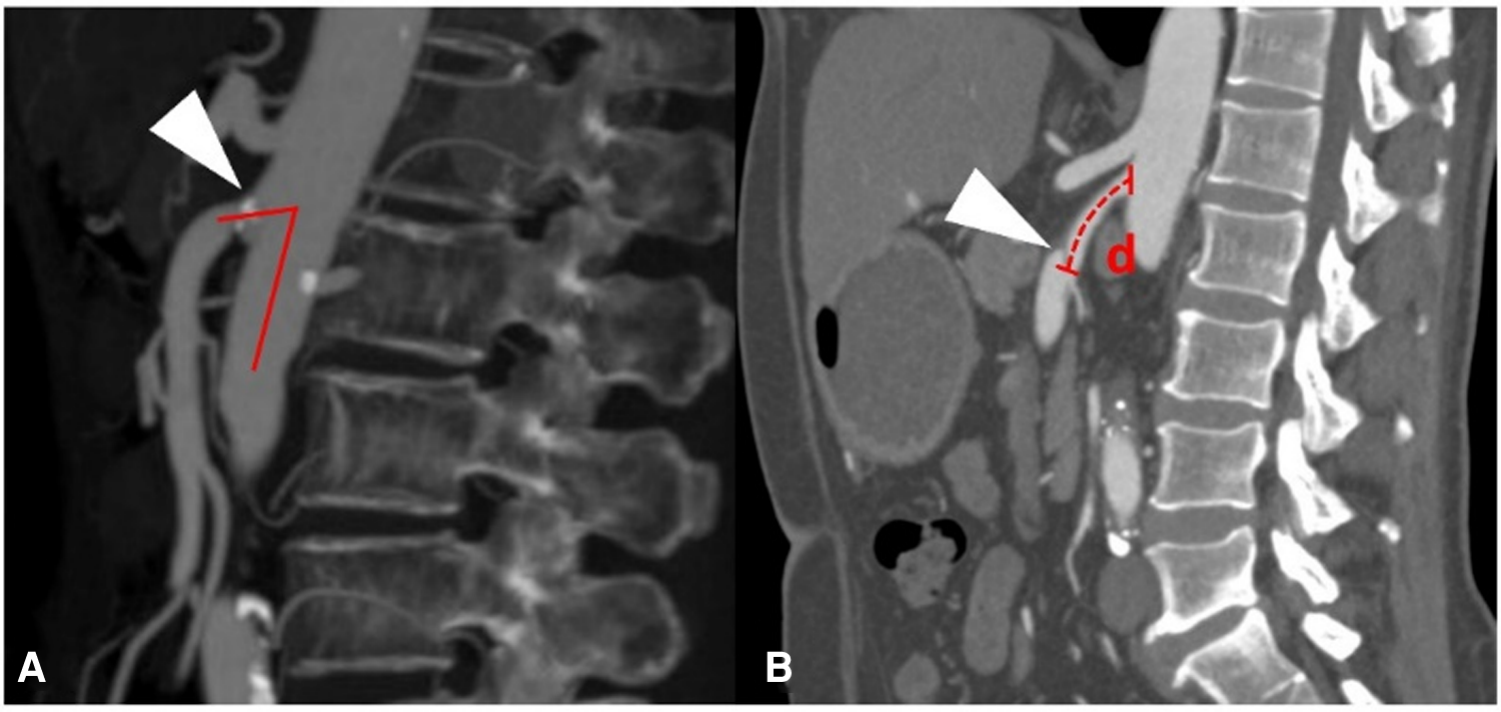
Measurements obtained using findings from computed tomography angiography. (**A**) Measurement of the aortomesenteric angle (red lines) and location of the superior mesenteric atherosclerotic plaque (white arrow). (**B**) Measurement of the distance (red *d*) from the root of the superior mesenteric artery to the origin of the superior mesenteric artery dissection (white arrow).

CT angiography (CTA) was performed on a 64-detector row scanner (Philips Core128, Rotterdam, The Netherlands). The scanning parameters were as follows: 120 kV; 312 mA; field of view, 362 × 362 cm; matrix, 512 × 512; and slice thickness, 0.5 mm. The locations of the plaque and dissection origins were assessed on the volume viewer using multiplanar reformatting and maximum intensity projections. The aortomesenteric angle was defined as the angle between the abdominal aorta major axis and the SMA ostium major axis (Figure 1A). The aortomesenteric angle and SMA-related distances were measured based on CTA findings (Figure 1B). All images were analyzed separately by 2 independent radiologists, both with >10 years' experience in vascular radiology. The measurements were repeated 20 days later, and the mean of the 2 sets of values was used for analysis.

### Model reconstruction and simulation

**Figure 2:**
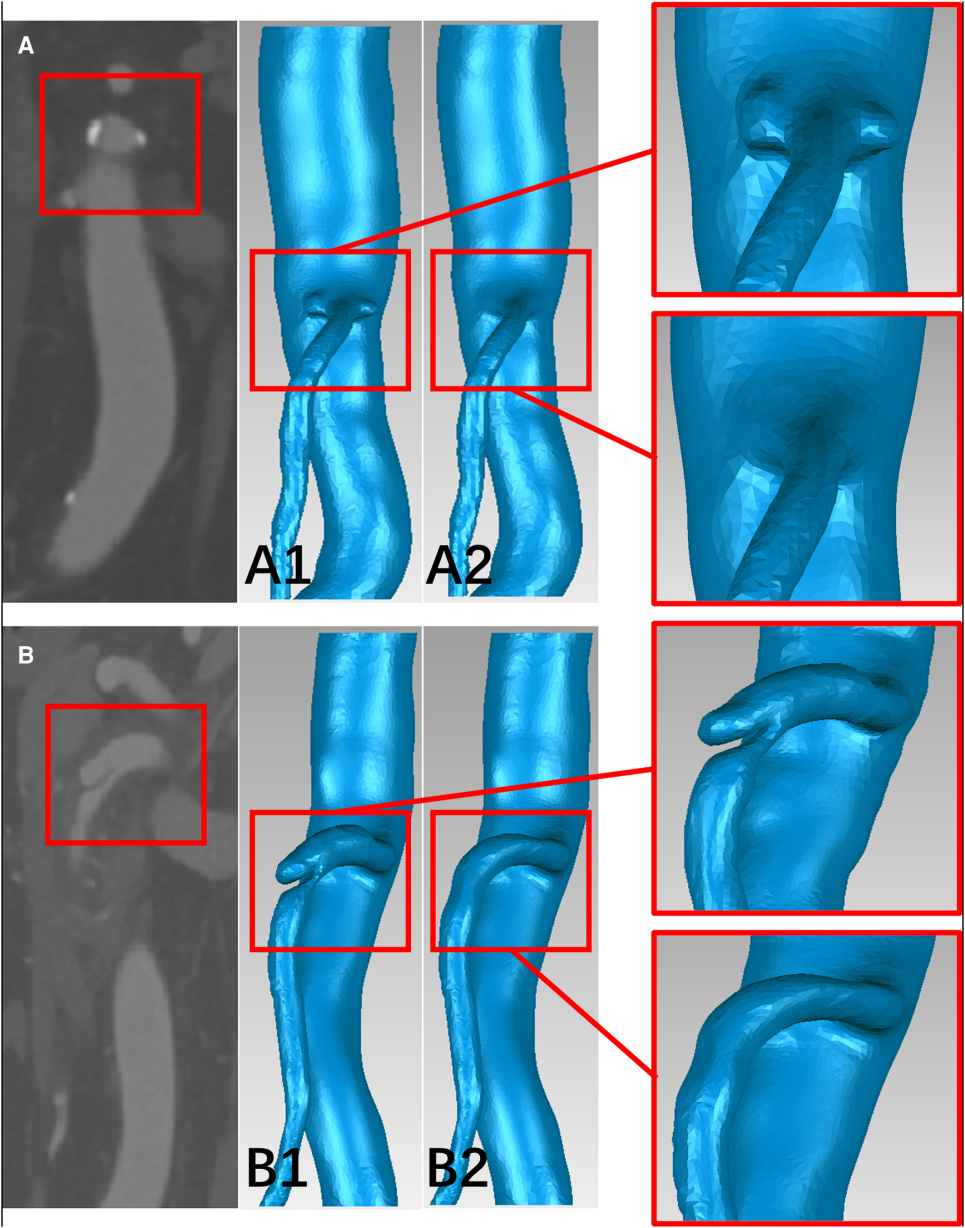
The schematic diagram of the progress of removing the plaques and dissections from the models. (**A,B**) CTA image of plaques at the root of the superior mesenteric artery (SMA) and dissection at the curved segment of the SMA. (**A1,B1**) The superior mesenteric atherosclerotic stenosis (SMAS) and superior mesenteric artery dissection (SMAD) models before repairing. (**A2,B2**) the SMA models before the onset of SMAS and SMAD.

The 3D surface of the SMA was abstracted from the CTA data using Mimics Research 19.0 (Materialise HQ, Louvain, Belgium). The models were then modified based on data from 60 patients, with SMAD (n = 30) and SMAS (n = 30), thirty-one SMAD patients who lack thin-slice CTA data or have poor image quality were excluded in the CFD analysis. At the same time, thirty SMAS patients meeting the including criteria were randomly included. For each model, dissections or plaques were removed and the main morphology features of the SMA trunk were retained to simulate the hemodynamic conditions in the SMA before the onset of SMAD or SMAS; this procedure was performed using Geomagic Wrap 2017 (Geomagic Studio, Raindrop, Research Triangle Park, NC, USA) and SolidWorks 2017 (Dassault Systemes SE, Paris, France) (Figure 2). The SMA models was divided into three segments: the root, the curved segment, and the distal segment. The root was identified as the region 0 to 5 mm away from the SMA ostium; the curved segment was identified as the region 15 to 30 mm away from the ostium; and the distal segment was identified as the region 40 to 55 cm away from the ostium.

**Figure 3:**
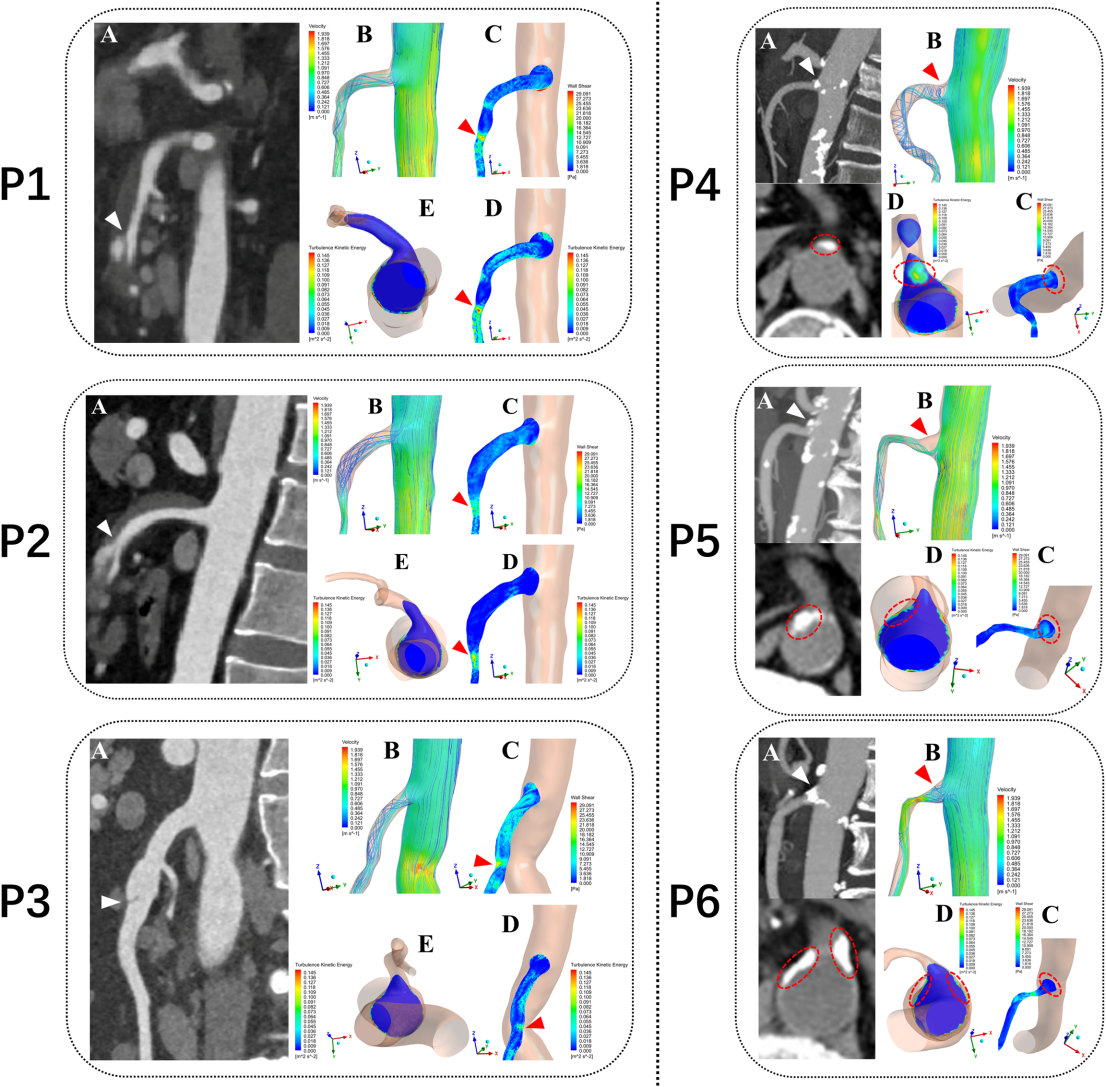
Computational fluid dynamics simulation outcomes from six typical patients. P1, P2 and P3 represent three patients with superior mesenteric artery dissection (SMAD), P4, P5 and P6 represent three patients with superior mesenteric atherosclerotic stenosis (SMAS). P1, P2 and P3: (**A**) Sagittal CT images showing location of the dissections (white arrows). P4, P5 and P6: (**A**) Sagittal and Axial CT images showing location of the plaques (white arrows). P1–P6: (**B**) Blood flow velocity (BFV) pattern of the superior mesenteric artery (SMA). (**C**) Wall shear stress (WSS) distribution at the wall of the SMA. (**D,E**) Turbulent kinetic energy (TKE) distribution at the wall of the superior mesenteric artery (SMA) (**E** in P1–P3) and cross-sectional plane at the SMA root (**E** in P1–P3 and **D** in P4–P6). red arrows and circles represent the hemodynamic abnormality regions.

Tetrahedral mesh was divided and the prism layer was created by using ICEM-CFD (Ansys, Canonsburg, PA, USA). Steady-state flow simulations corresponding to the flow conditions at the patient-specific systolic peak were performed using the CFD solver in Fluent (Ansys, Canonsburg, PA, USA) by solving a *k-ω* SST transition turbulence model (13). Blood was treated as an in compressible Newtonian fluid with a density of 1,045 kg/m^3^ and a dynamic viscosity of 0.00365 Pa·s (14). For the initial boundary conditions at the vascular openings, we assumed a constant velocity inlet of the patient-specific peak systolic blood flow velocity (cross-section of the abdominal aorta, 5 cm above the SMA ostium) and zero pressure outlet at SMA and cross-section of the abdominal aorta above the common iliac artery for the geometry were applied (15). The mean values of turbulent kinetic energy (TKE), blood flow velocity (BFV), and wall shear stress (WSS) in the three segments of the SMA were then calculated and compared in the 60 patients models. The nephograms of the hemodynamic characteristics in six of them was then displayed: SMAD (P1, P2 and P3), SMAS (P4, P5 and P6) (Figure 3).

### Histopathologic analysis

To explore precursor histopathologic changes occurring in the SMA wall, we obtained SMA specimens from 10 cadavers (all males, aged 45–65 years) with noncardiovascular causes of death. Individuals who died from other cardiovascular-related diseases but experienced SMAS or SMAD before death were excluded. SMA sections were stained with hematoxylin and eosin to measure the thickness of each layer of vascular wall and to analyze the cell composition and degenerative changes. The tissues of the SMA were also divided into root, curved segment, and distal segment same as the model reconstruction method. Each of the three segments was also dissected into posterior and anterior walls. The collagen and lamellar microstructure in the three segments of the SMA was analyzed using scanning electron microscopy (SEM).

### Statistical analysis

SPSS 26.0 (SPSS Inc., Chicago, IL, USA) was used for statistical analysis. Kolmogorov-Smirnov test method was used for the test of normality. Normally distributed continuous measurements were calculated as mean ± standard deviation. Patient characteristics, lesion locations, and morphologic features of the SMA were compared between patients with SMAS and those with SMAD using independent sample *t*-tests and chi-square tests or Fisher exact test (for small samples or highly imbalanced table cells). The thickness of each layer and the mean values of WSS, TKE, and BFV in the different segments of the SMA were compared using independent sample *t*-tests. Statistical significance was set at *p *< .05.

## Results

### Patients

A total of 124 consecutive patients with SMAS (66 men, 58 women; mean age, 71.5 ± 10.3 years; range, 31–96 years) and 61 consecutive patients with SMAD (51 men, 10 women; mean age, 56.1 ± 9.1 years; range, 35–83 years) were included in this analysis. Table 1 summarizes the clinical characteristics of study patients.

**Table 1:** Clinical characteristics of study patients.

Characteristic	Patients with SMAS (*n* = 124)	Patients with SMAD (*n* = 61)	*p* value
Age, y	71.5 ± 10.3	56.1 ± 9.1	<.001
Male	66 (53.2)	51 (83.6)	<.001
Body mass index	23.7 ± 3.0	24.6 ± 2.7	.053
Smoking history	44 (35.5)	24 (39.3)	.609
Hypertension	71 (57.3)	30 (49.2)	.300
Diabetes mellitus	38 (30.7)	7 (11.5)	.004
Hyperlipidemia	41 (33.1)	21 (34.4)	.854
High uric acid level	14 (11.3)	4 (6.6)	.307

Data are presented as *n* (%) or mean ± standard deviation. SMAS, superior mesenteric atherosclerotic stenosis; SMAD, superior mesenteric artery dissection.

### CT findings

The mean aortomesenteric angle was larger in patients with SMAD than in patients with SMAS (62.5° ± 20.4° vs. 56.3° ± 17.2°; *p* = .031). The distance from the root of the SMA to the dissection origin in patients with SMAD was longer than the distance from the root of the SMA to the plaque in patients with SMAS (21.4 ± 11.4 mm vs. 1.3 ± 4.4 mm; *p* < .001). Of note, 88.7% of plaques were located at the SMA root, whereas 96.7% of dissection origins were located at the anterior wall of the SMA. Table 2 summarizes the information regarding these plaque and dissection locations.

**Table 2:** CT findings in study patients.

Characteristic	Patients with SMAS (*n* = 124)	Patients with SMAD (*n* = 61)	*p* value
Aortomesenteric angle, °	56.3 ± 17.2	62.5 ± 20.4	.031
Distance, mm	1.3 ± 4.4	21.4 ± 11.4	<.001
Location of plaque or dissection			
Anterior	68 (54.8)	59 (96.7)	<.001
Anterior middle	19 (15.3)	33 (54.1)	<.001
Anterior and left	23 (18.6)	14 (23.0)	.482
Anterior and right	15 (12.1)	12 (19.7)	.170
Anterior and bilateral	11 (8.9)	0 (0)	.017
Side	37 (29.8)	1 (1.6)	<.001
Left	17 (13.7)	0 (0)	.002
Right	11 (8.9)	1 (1.6)	.108
Bilateral	9 (7.3)	0 (0)	.031
Posterior	14 (11.3)	1 (1.6)	.023
Posterior middle	4 (3.2)	1 (1.6)	>.990
Posterior and left	7 (5.7)	0 (0)	.098
Posterior and right	3 (2.4)	0 (0)	.552
Posterior and bilateral	0 (0)	0 (0)	N/A
Round	5 (4.0)	0 (0)	.173

Data are presented as *n* (%) or mean ± standard deviation. SMAS, superior mesenteric atherosclerotic stenosis; SMAD, superior mesenteric artery dissection; N/A, not available. Distance = distance from the root of the superior mesenteric artery to the location of the plaque or dissection origin. Round = All directions around the SMA wall.

### Hemodynamic factors

**Figure 4:**
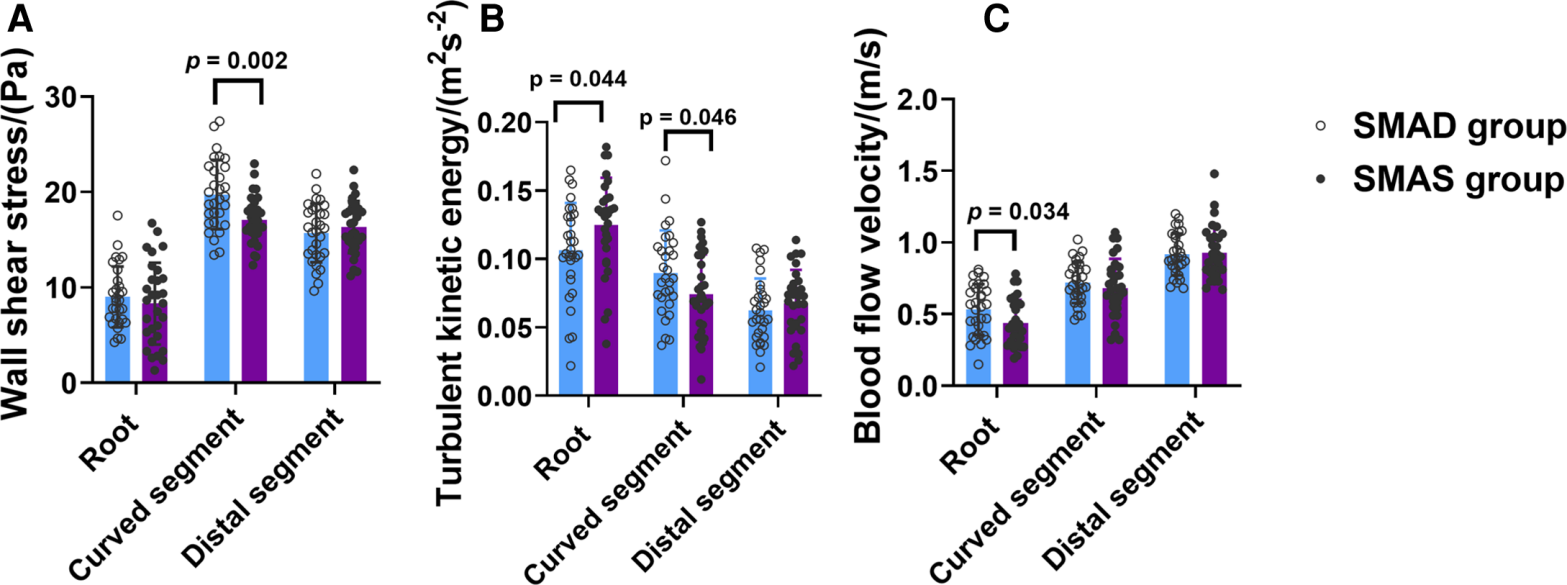
The comparison of hemodynamic parameters, including Wall shear stress (WSS) (**A**), turbulent kinetic energy (TKE) (**B**), and blood flow velocity (BFV) (**C**), in the three segments (root, curved and distal segment) of the superior mesenteric artery (SMA) in the superior mesenteric artery dissection (SMAD) and superior mesenteric atherosclerotic stenosis (SMAS) groups.

The SMAD group had higher WSS than the SMAS group in the curved segment (19.74 ± 3.63 pa vs. 17.09 ± 2.48 pa; *p* = .002); and the WSS in the curved segment was higher than in the other two segments in both groups. Compared with the SMAD group, the SMAS group had higher TKE in the root (0.125 ± 0.035 m^2^s^−2^ vs. 0.106 ± 0.035 m^2^s^−2^; *p* = .044) but lower TKE in the curved segment (0.090 ± 0.031 m^2^s^−2^ vs. 0.074 ± 0.029 m^2^s^−2^; *p* = 0.046); there was no difference between groups in the TKE of the distal segment. The TKE values in the root and the curved segment were higher than in the distal segment in both groups. The BFV in patients with SMAS was lower than in patients with SMAD in root (0.44 ± 0.16 m/s vs. 0.53 ± 0.18 m/s; *p* = 0.034); there was no difference between the groups in the BFV values in the other segments (Figure 4).

In the 6 SMA models (P1 to P6), the regions of high TKE were mainly distributed around the SMA root and almost overlapped with the low BFV vortex regions. In the curved segment of the SMA, the regions of high TKE and BFV coexisted below the anterior convex wall of the SMA. The distribution of high WSS was consistent with the dissection origin (P1 to P3). The high TKE and low BFV regions at the SMA root were consistent with the location of plaques (P4 to P6). An annular low WSS zone appeared at the root of the SMA, whereas the WSS level in the anterior convex wall of the SMA curve was substantially increased. Flow recirculation was observed below the anterior and side walls of the SMA root (Figure 3).

### Histopathology

**Figure 5:**
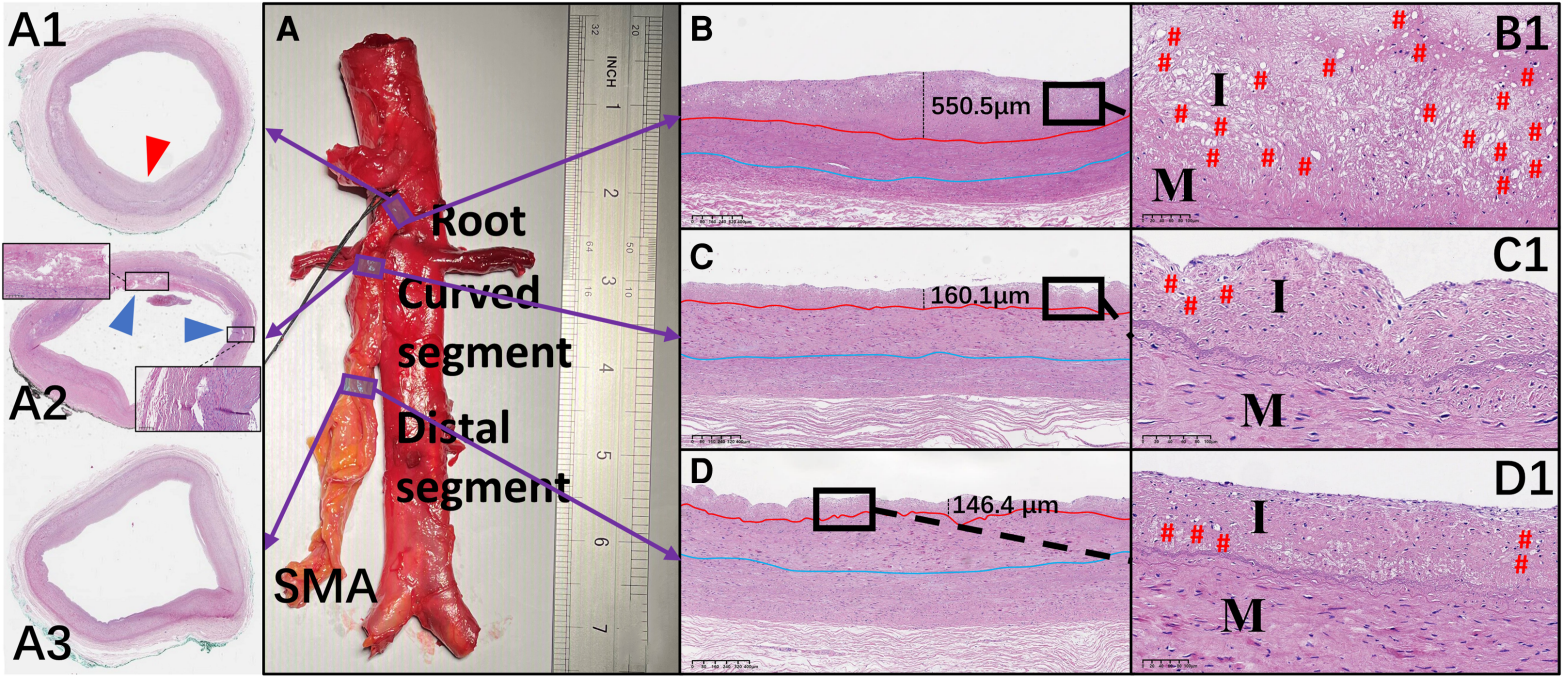
Representative images of hematoxylin and eosin–stained sections of the superior mesenteric artery (SMA) wall and comparison of the mean thickness of each layer of the vascular wall in the different regions of the SMA. (**A–D** and **A1–D1**) Hematoxylin and eosin staining of the wall located at the root (**A1**), curved segment (**A2**), and distal end (**A3**) of the SMA. Thickening of the intima (red arrow) and translamellar mucoid extracellular matrix accumulation (blue arrows) can be observed. (**B–D**) The thickness of each intima is shown. (**B1–D1**) Local magnification of the images in **B–D**. # represents the location of foam cells. I, intima; M, media.

Hematoxylin and eosin–stained sections are shown in Figure 5. Crescent-shaped intimal thickening was observed at the root of the SMA (Figure 5A1). Foam cells with lipid droplets in the cytoplasm were more common in the intima at the SMA root than in other segments (Figures 5B1–D1). Translamellar mucoid extracellular matrix accumulation breaking through the lamellar units was observed in the media of the anterior wall at the curved segment of the SMA (Figure 5A2). At the distal segment of the SMA, the structure of the circumferential arterial wall was uniform, with no obvious degenerative changes (Figure 5A3).

**Figure 6:**
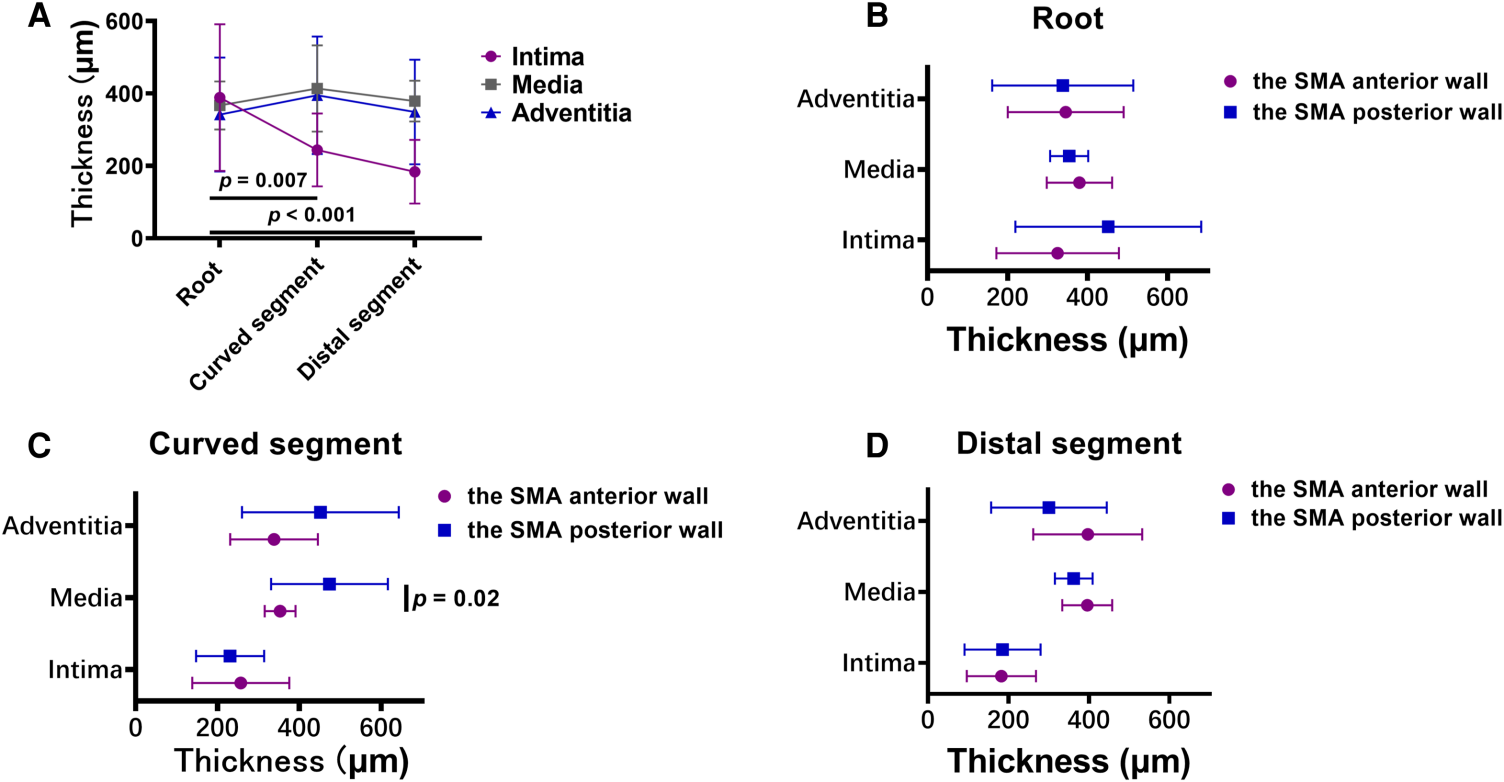
(**A**) broken-line graphs of the mean value of the thickness of each layer of the vascular wall in the three superior mesenteric artery (SMA) segments. (**B–D**) The thickness of each layer of the vascular wall in the root (**B**), curved segment (**C**), and distal segment (**D**) of the SMA.

The mean thickness of the intima at the root of the SMA (388.5 ± 202.3 µm) was greater than that in the curved (243.8 ± 100.5 µm; *p *= .007) and distal (183.7 ± 88.0 µm; *p* < .001) segments. However, there was no significant difference in the mean thickness of the other 2 layers across the 3 locations of the SMA. In the curved segment of the SMA, the mean thicknesses of the intima (256.8 ± 118.4 µm), media (353.1 ± 37.6 µm), and adventitia (338.2 ± 107.2 µm) at the anterior wall were lower than the mean thicknesses of the intima (230.7 ± 83.2 µm; *p* = .58), media (473.7 ± 142.8 µm; *p* = .02), and adventitia (451.4 ± 191.8 µm; *p *= .12) at the posterior wall. There was no significant difference between the anterior and posterior walls in the mean thicknesses of the 3 layers at the other 2 locations of the SMA (Figures 6A–D).

**Figure 7:**
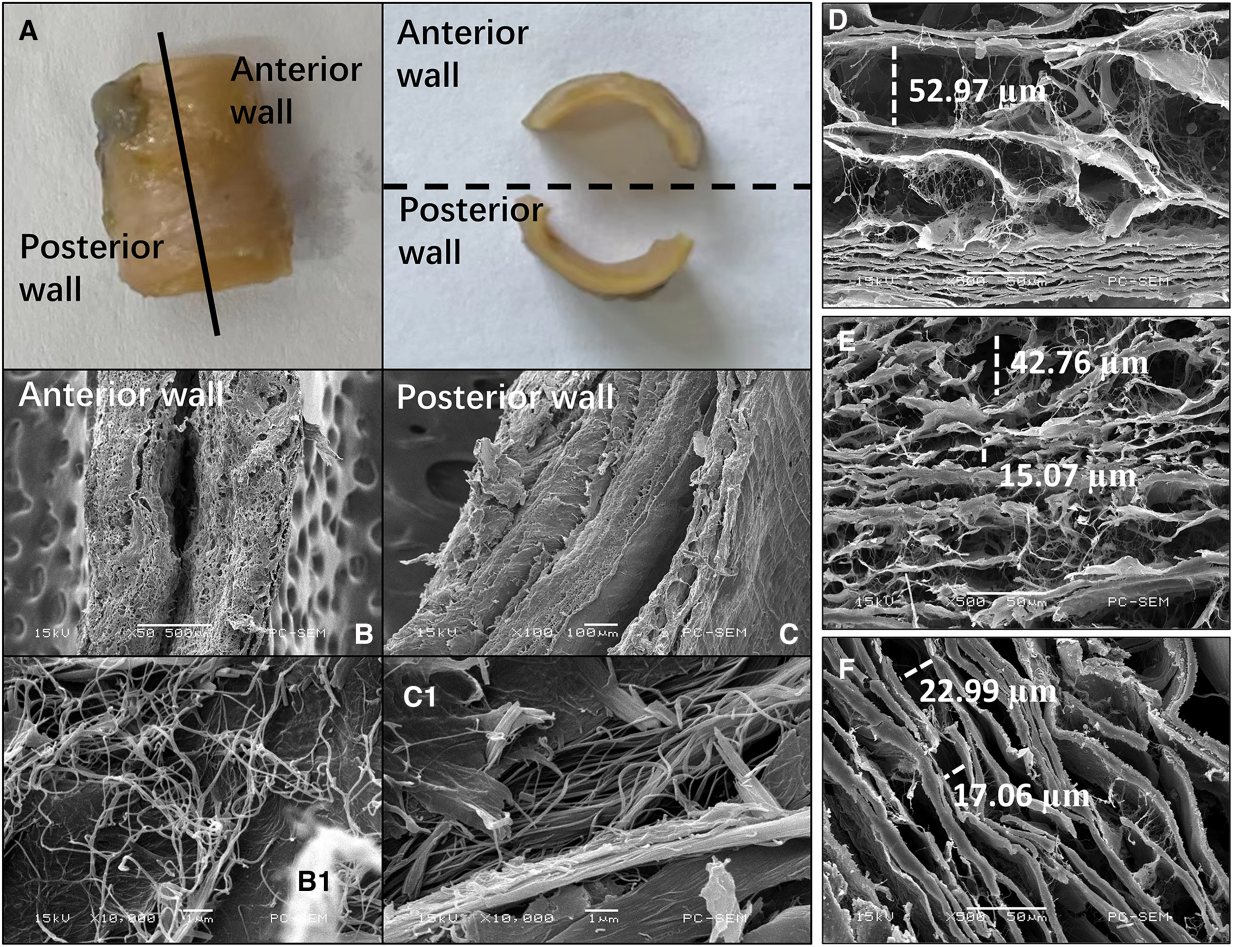
Images of the superior mesenteric artery (SMA) wall from an SMA specimen. (**A**) SMA specimen divided into anterior and posterior walls. (**B,C**) Cross-sectional scanning electron microscopy images of the anterior (**B**) and posterior (**C**) wall of the curved segment of the SMA. (**B1, C1**) Collagen microstructure skeletonization images showing the area between the intima and media of the anterior (**B1**) and posterior (**C1**) walls in the curved segment of the SMA. (**D–F**) Scanning electron microscopy images showing the lamellar structure between the intima and media in the root (**D**), curved segment (**E**), and distal segment (**F**) of the SMA. The widths of some gaps have been marked.

Each segment of the SMA was separated into anterior and posterior walls (Figure 7A). The gaps in the lamellar structure were larger in the SMA root than in the curved and distal segments (Figures 7D–F). In the curved segment, the delamination of each layer was not obvious in the anterior wall (Figure 7B), whereas a clear 3-layer structure could be seen in the posterior wall (Figure 7C). Microstructure analysis of the collagen fibers between the intima and media showed that the fibers in the anterior wall were of uneven thickness and were interlaced in a disorderly manner (Figure 7B1), whereas the fibers in the posterior wall had a uniform thickness, with all fibers generally positioned in the same direction (Figure 7C1). In the other two segments of the SMA, the above phenomenon was not obvious.

## Discussion

In this study, we found that SMAS always occurred near the SMA root and most commonly in the anterior wall (54.8%), whereas the origin of SMAD was most commonly seen in the anterior wall of the curved section of the SMA (96.7%). Through CFD analysis, we found that high TKE regions were distributed in the root of the SMA and the anterior wall of the curved portion of the SMA, whereas regions of vortex, low flow velocity, and low WSS were seen near the plaque at the root of the SMA. Plaques were commonly observed at the aortic bifurcation. Previous studies have demonstrated associations between hemodynamic abnormalities and high concentrations of low-density lipoprotein (LDL) and lower levels of endothelial nitric oxide synthase gene expression, both of which are necessary for the formation of plaque (16–19). In the current study, regions of high flow velocity and high WSS were observed at the curved segment of the SMA, near the origin of the dissection. Previous studies have linked abnormally elevated WSS to medial degeneration, loss of smooth muscle cells, and internal elastic lamina fracture, factors associated with dissection and aneurysm (20, 21). The results from our current study support these findings and confirm the relationship between high WSS and the occurrence of SMAD demonstrated in previous research (18).

Histopathologic analysis in this study demonstrated that more foam cells were present in the intima of the SMA root and that the intima was thicker at the SMA root than in the other SMA segments. Based on the SEM images we obtained, we believe that the larger gaps in the lamellar structure in the SMA root facilitate the migration and multiplication of lipids under the intima, with the rotation and stagnation of blood flow at the root of the SMA also aggravating this process. These factors explain the high incidence of SMAS at the SMA root (22).

In the curved segment of the SMA, the anterior wall was found to be significantly thinner than the posterior wall. Previous research has shown that thinning of the anterior wall is closely related to the loss of smooth muscle cells (23). The current study also demonstrated translamellar mucoid extracellular matrix accumulation breaking across laminar units in the anterior wall. This reduces the strength of the wall and is therefore considered an important index for the evaluation of medial degeneration (24). In our study, the microstructure of collagen fibers was also found to be substantially different between the anterior and posterior walls; the collagen fibers in the anterior wall had more cross-kinking and were of uneven thickness, whereas the collagen fibers in the posterior wall were generally uniform in thickness and direction. Previous research has shown that there are more cross-linked collagen fibers in the wall of aneurysms than in healthy arteries, and an increase in cross-linking reflects weakening of the renewal ability of collagen fibers (25, 26). In the extracellular matrix, collagen fibers provide resistance to dissection and rupture (23), and so weakness in these fibers can allow for the occurrence of SMAD.

The complex mechanical loads from blood flow can also cause changes in the function and structure of the vascular wall either directly or indirectly. In the intima, blood flow may directly cause realignment, stiffness, and dysfunction of endothelial cells (27, 28). Smooth muscle cells and extracellular matrix in the media and adventitia are not directly exposed to blood flow, but blood flow can still indirectly influence these areas through the signaling molecules produced by endothelial cells (29). The media and adventitia may also be affected by the interstitial flow between the smooth muscle cells and extracellular matrix (30), which may increase migration, phenotypic changes, and apoptosis of smooth muscle cells (31) and reduce production of the surrounding extracellular matrix (30, 32), thus affecting the elasticity, tensile stiffness, and strength of the wall. Recent investigations have demonstrated that elevated WSS may be a predictor of media degeneration, which causes the thinning of media and reduction of elastin and smooth muscle cells (33, 34) and eventually leads to the occurrence of dissection or aneurysm (23). Mechanical stretch and pressure also affect the vascular wall. However, the cell signaling pathway involved in mechanical stimulation of the intima, media, and adventitia is still unclear (21).

This study had several limitations. First, the number of SMA specimens in this study was limited, so quantitative histopathologic studies with larger sample sizes are needed. Second, although we were able to evaluate the vascular wall structure in various locations of the SMA, the relationship between changes in tissue structure and biomechanical properties needs to be verified using other testing methods such as biaxial biomechanical testing. Third, although we assessed the intact portion between matrix fissures as far as possible when observing collagen fibers on SEM, the process of manually splitting the intima and media inevitably causes damage to the natural collagen fiber structure. Fourth, the histologic analysis in this study was based on prodromal changes of the vascular wall in healthy individuals; tissues obtained from patients with SMAS or SMAD may be more reliable for analysis. Fifth, the progress of removing the plaques and dissections from the models was manual, which may cause restoration distortion in some cases. Finally, steady-state flow simulations were performed in our CFD analysis. Personalized CFD simulations or 4D flow magnetic resonance imaging examinations should be performed in larger studies to further verify the link between hemodynamic abnormalities and lesion occurrence sites, and more hemodynamic indices (eg, oscillatory shear index) should be included in future studies.

In conclusion, this study demonstrated that the locations of SMAS and SMAD differ based on hemodynamic factors. Further, histopathologic analysis demonstrated that early atherosclerotic changes and local vascular wall degeneration can be seen near SMAS and SMAD. These findings suggest that various hemodynamic factors and vascular wall characteristics play an important role in the occurrence of SMAS and SMAD.

## Data Availability

The original contributions presented in the study are included in the article/supplementary material, further inquiries can be directed to the corresponding author/s.

## A summary statement

Hemodynamic factors play a different but equally important role in the pathogenesis and occurrence of SMAS and SMAD.

## Key results

124 patients diagnosed with SMAS and 61 patients diagnosed with SMAD were included. Abnormal hemodynamic regions in the root and anterior wall of the curved segment of the superior mesenteric artery, and corresponding histopathologic changes were found, which were in accordance with frequent incidence location of SMAS and SMAD, respectively.

## References

[1] Lehtimäki TT Kärkkäinen JM Saari P Manninen H Paajanen H Vanninen R. Detecting acute mesenteric ischemia in CT of the acute abdomen is dependent on clinical suspicion: review of 95 consecutive patients. Eur J Radiol. (2015) 84:2444–53. 10.1016/j.ejrad.2015.09.006 26413771

[2] Luan JY Guan X Li X Wang CM Li TR Zhang L Isolated superior mesenteric artery dissection in China. J Vasc Surg. (2016) 63:530–6. 10.1016/j.jvs.2015.09.047 26597665

[3] Günenç Beşer C Karcaaltıncaba M Çelik HH Başar R. The prevalence and distribution of the atherosclerotic plaques in the abdominal aorta and its branches. Folia Morphol (Warsz). (2016) 75:364–75. 10.5603/FM.a2016.0005 26821603

[4] Naeem A Nasim N Ihsan U Masood A. A morphological study of celiac, superior mesenteric and inferior mesenteric arteries in atherosclerosis. J Ayub Med Coll Abbottabad. (2012) 24:18–21. PMID: . 24397043

[5] Dou L Tang H Zheng P Wang C Li D Yang J. Isolated superior mesenteric artery dissection: cTA features and clinical relevance. Abdom Radiol (NY). (2020) 45:2879–85. 10.1007/s00261-019-02171-4 31401677

[6] Tomita K Obara H Sekimoto Y Matsubara K Watada S Fujimura N Evolution of computed tomographic characteristics of spontaneous isolated superior mesenteric artery dissection during conservative management. Circ J. (2016) 80:1452–9. 10.1253/circj.CJ-15-1369 27118619

[7] Park J Lee JM Koo BK Choi G Hwang D Rhee TM Relevance of anatomical, plaque, and hemodynamic characteristics of non-obstructive coronary lesions in the prediction of risk for acute coronary syndrome. Eur Radiol. (2019) 29:6119–28. 10.1007/s00330-019-06221-9 31025066

[8] Park J Lee JM Koo BK Choi G Hwang D Rhee TM Inference from clinical and fluid dynamic studies about underlying cause of spontaneous isolated superior mesenteric artery dissection. J Vasc Surg. (2011) 53:80–6. 10.1016/j.jvs.2010.07.055 20855179

[9] Stone PH Coskun AU Croce KJ. Evolving insights into the role of local shear stress in late stent failure from neoatherosclerosis formation and plaque destabilization. Int J Cardiol. (2018) 272:45–6. 10.1016/j.ijcard.2018.08.016 30126653

[10] Björck M Koelemay M Acosta S Goncalves Bastos F Kölbel T Kolkman JJ Editor's choice—management of the diseases of mesenteric arteries and veins: clinical practice guidelines of the European society of vascular surgery (ESVS). Eur J Vasc Endovasc Surg. (2017) 53:460–510. 10.1016/j.ejvs.2017.01.010 28359440

[11] Fleischmann D. Multiple detector-row CT angiography of the renal and mesenteric vessels. Eur J Radiol. (2003) 45:S79–87. 10.1016/S0720-048X(02)00364-9 12598031

[12] Jia Z Huang Y Shi H Tang L Shi H Qian L Comparison of CTA and DSA in the diagnosis of superior mesenteric artery dissecting aneurysm. Vascular. (2018) 26:346–51. 10.1177/1708538117739540 29105573

[13] Goubergrits L Vellguth K Obermeier L Schlief A Tautz L Bruening J CT-Based analysis of left ventricular hemodynamics using statistical shape modeling and computational fluid dynamics. Front Cardiovasc Med. (2022) 9:901902. 10.3389/fcvm.2022.901902 35865389 PMC9294248

[14] Jia Z Mei J Ding W Zhao X Gong W Yu H The pathogenesis of superior mesenteric artery dissection: an in-depth study based on fluid-structure interaction and histology analysis. Comput Methods Programs Biomed. (2022) 226:107187. 10.1016/j.cmpb.2022.107187 36279640

[15] Zhang M Li Y Zhao X Verrelli DI Chong W Ohta M Haemodynamic effects of stent diameter and compaction ratio on flow-diversion treatment of intracranial aneurysms: a numerical study of a successful and an unsuccessful case. J Biomech. (2017) 58:179–86. 10.1016/j.jbiomech.2017.05.001 28576622

[16] Ameenuddin M Anand M. A mixture theory model for blood combined with low-density lipoprotein transport to predict early atherosclerosis regions in idealized and patient-derived abdominal aorta. J Biomech Eng. (2020) 142:101008. 10.1115/1.4047426 32507886

[17] Samady H Eshtehardi P McDaniel MC Suo J Dhawan SS Maynard C Coronary artery wall shear stress is associated with progression and transformation of atherosclerotic plaque and arterial remodeling in patients with coronary artery disease. Circulation. (2011) 124:779–88. 10.1161/CIRCULATIONAHA.111.021824 21788584

[18] Stone PH Saito S Takahashi S Makita Y Nakamura S Kawasaki T Prediction of progression of coronary artery disease and clinical outcomes using vascular profiling of endothelial shear stress and arterial plaque characteristics: the prediction study. Circulation. (2012) 126:172–81. 10.1161/CIRCULATIONAHA.112.096438 22723305

[19] Vergallo R Papafaklis MI Yonetsu T Bourantas CV Andreou I Wang Z Endothelial shear stress and coronary plaque characteristics in humans: combined frequency-domain optical coherence tomography and computational fluid dynamics study. Circ Cardiovasc Imaging. (2014) 7:905–11. 10.1161/CIRCIMAGING.114.001932 25190591

[20] Bollache E Guzzardi DG Sattari S Olsen KE Di Martino ES Malaisrie SC Aortic valve-mediated wall shear stress is heterogeneous and predicts regional aortic elastic fiber thinning in bicuspid aortic valve-associated aortopathy. J Thorac Cardiovasc Surg. (2018) 156:2112–2120.e2. 10.1016/j.jtcvs.2018.05.095 30060930 PMC6242768

[21] Humphrey JD Schwartz MA Tellides G Milewicz DM. Role of mechanotransduction in vascular biology: focus on thoracic aortic aneurysms and dissections. Circ Res. (2015) 116:1448–61. 10.1161/CIRCRESAHA.114.304936 25858068 PMC4420625

[22] Mazzi V De Nisco G Hoogendoorn A Calò K Chiastra C Gallo D Early atherosclerotic changes in coronary arteries are associated with endothelium shear stress contraction/expansion variability. Ann Biomed Eng. (2021) 49:2606–21. 10.1007/s10439-021-02829-5 34324092 PMC8455396

[23] Michel JB Jondeau G Milewicz DM. From genetics to response to injury: vascular smooth muscle cells in aneurysms and dissections of the ascending aorta. Cardiovasc Res. (2018) 114:578–89. 10.1093/cvr/cvy006 29360940 PMC6658716

[24] Halushka MK Angelini A Bartoloni G Basso C Batoroeva L Bruneval P Consensus statement on surgical pathology of the aorta from the society for cardiovascular pathology and the association for European cardiovascular pathology: iI. Noninflammatory degenerative diseases—nomenclature and diagnostic criteria. Cardiovasc Pathol. (2016) 25:247–57. 10.1016/j.carpath.2016.03.002 27031798

[25] Lindeman JH Ashcroft BA Beenakker JW van Es M Koekkoek NB Prins FA Distinct defects in collagen microarchitecture underlie vessel-wall failure in advanced abdominal aneurysms and aneurysms in marfan syndrome. Proc Natl Acad Sci USA. (2010) 107:862–5. 10.1073/pnas.0910312107 20080766 PMC2818895

[26] Tsamis A Krawiec JT Vorp DA. Elastin and collagen fibre microstructure of the human aorta in ageing and disease: a review. J R Soc Interface. (2013) 10:20121004. 10.1098/rsif.2012.1004 23536538 PMC3645409

[27] Ho RX Tahboub R Amraei R Meyer RD Varongchayakul N Grinstaff M The cell adhesion molecule IGPR-1 is activated by and regulates responses of endothelial cells to shear stress. J Biol Chem. (2019) 294:13671–80. 10.1074/jbc.RA119.008548 31341021 PMC6746441

[28] Mehta V Pang KL Rozbesky D Nather K Keen A Lachowski D The guidance receptor plexin D1 is a mechanosensor in endothelial cells. Nature. (2020) 578:290–5. 10.1038/s41586-020-1979-4 32025034 PMC7025890

[29] Lu D Kassab GS. Role of shear stress and stretch in vascular mechanobiology. J R Soc Interface. (2011) 8:1379–85. 10.1098/rsif.2011.0177 21733876 PMC3163429

[30] Shi ZD Tarbell JM. Fluid flow mechanotransduction in vascular smooth muscle cells and fibroblasts. Ann Biomed Eng. (2011) 39:1608–19. 10.1007/s10439-011-0309-2 21479754 PMC3184546

[31] Taylor B ES Appelboom G Zilinyi R Goodman A Chapel D LoPresti M Role of the complement cascade in cerebral aneurysm formation, growth, and rupture. Neurol-Neuroimmunol. (2015) 2:93–101. 10.4103/2347-8659.154888

[32] Stegemann JP Hong H Nerem RM. Mechanical, biochemical, and extracellular matrix effects on vascular smooth muscle cell phenotype. J Appl Physiol. (2005) 98:2321–7. 10.1152/japplphysiol.01114.2004 15894540

[33] Salmasi MY Pirola S Sasidharan S Fisichella SM Redaelli A Jarral OA High wall shear stress can predict wall degradation in ascending aortic aneurysms: an integrated biomechanics study. Front Bioeng Biotechnol. (2021) 9:750656. 10.3389/fbioe.2021.750656 34733832 PMC8558434

[34] Staarmann B Smith M Prestigiacomo CJ. Shear stress and aneurysms: a review. Neurosurg Focus. (2019) 47:E2. 10.3171/2019.4.FOCUS19225 31261124

